# Grand Challenges in Ocular Inflammatory Diseases

**DOI:** 10.3389/fopht.2022.756689

**Published:** 2022-02-17

**Authors:** Heping Xu, Narsing A. Rao

**Affiliations:** ^1^ The Wellcome-Wolfson Institute for Experimental Medicine, Queen’s University Belfast, Belfast, United Kingdom; ^2^ Aier Institute of Optometry and Vision Science, Changsha, China; ^3^ Department of Ophthalmology, USC-Roski Eye Institute, Los Angeles, CA, United States; ^4^ Keck School of Medicine, University of Southern California, Los Angeles, CA, United States

**Keywords:** inflammation, microbiome, blood ocular barrier, aging, degeneration, omics technology, machine learning, artificial intelligence

## Introduction

There are two categories of ocular inflammatory disease, inflammation-driven diseases and pathological changes/degenerative disorders with significant inflammatory components. Examples of the former include keratitis, conjunctivitis, and uveitis, and inflammation is the primary cause of the disease. Dry eye syndrome (DES), glaucoma, diabetic retinopathy (DR), and age-related macular degeneration (AMD) are typical examples of inflammation-related ocular degenerations. Although the initial triggers are not immune-related, chronic inflammation plays an important role in the initiation, progression and outcome of these disorders.

The clinical diagnosis and treatment of ocular inflammatory disorders are based on the anatomic location of the inflammation, as well as how the anatomic site plays a role in dissecting and understanding the pathogenesis of the inflammatory process. Based on the anatomic location, ocular inflammations are broadly grouped as (a) those involving conjunctiva, cornea and sclera as extraocular inflammations, and (b) those involving intraocular structures, called intraocular inflammations, where the uvea and the retina are the main sites of inflammation. Uveal inflammation can be limited to the iris and the ciliary body, the pars plana region or the choroid. They are recognized as anterior, intermediate, and posterior uveitis respectively. The inflammation involving the entire uveal tract is labelled as pan-uveitis.

## Inflammation-Driven Ocular Diseases

The uveitides can be caused by inflammation involving primarily uveal tissue. The uvea can be secondarily affected by inflammations of the lens, retina, optic nerve, sclera and cornea. Histologically, the uveitis reveals features of acute non-granulomatous or chronic inflammation of either granulomatous or non-granulomatous. Such histologic changes can reflect underlying immune-pathogenicity of non-infectious and infectious uveitides. These histologic variations are recognized by clinical examinations as well as an initiation of tailored laboratory investigations that can establish the etiologic diagnosis of uveitis in humans.

The uveitides can be of infectious etiology or autoimmune mediated. Globally, uveitis is a significant cause of blindness. In the U.S. it is estimated to be responsible for around 10-15 percent of legal blindness (1). The estimate of blindness can be higher in developing countries due to infectious causes.

In patients with infectious uveitis, the inflammatory process is also driven by innate and adaptive immunity directed toward eliminating the infectious agent. Such an immune process can prolong ocular inflammation and cause tissue damage as noted in tuberculous uveitis. Release of sequestered tissue antigens from the damaged tissues can initiate autoimmune inflammation. The trigger for the autoimmunity can derive from molecular mimicry of shared antigens of the infectious agent with the tissue antigens, adjuvant effects of the infectious agent and can also be formed by standard activation or epitope spreading (2). Such infectious and immune mediated inflammation complicates the treatment of infectious uveitis with antimicrobials to combat the infection, as well as the use of immunosuppressive agents to minimize immune driven inflammation and subsequent tissue damage. Use of immunomodulatory agents can enhance infectious processes, resulting in prolonged/chronic and recurrent inflammation, associated tissue damage and the sequelae of such inflammation, leading to the development of cataract, glaucoma and retinal damage. Thus, balancing elimination of the infectious agent and minimizing or preventing immune mediated prolonged inflammation or autoimmunity is a challenge. A clear understanding in eliminating the infectious agent and minimizing tissue damage from the infectious agent and immune process is required to prevent vision loss in infectious uveitides.

Usually, the incidence and prevalence of infectious uveitis represents the prevalence of endemic diseases of a region. A clear understanding of endemic diseases in a geographic location could enhance early clinical diagnosis and appropriate antimicrobial interventions. Current diagnosis of infectious uveitides requires a big data analysis of endemic diseases and their spread to non-endemic countries. In the United States, a recent big data study using nationwide medical claims revealed hitherto unknown higher incidences of infectious uveitis, stating that overall age increased the risk of infections uveitis significantly for each decade over the age of 18 years (1). Such data indicates older individuals are more prone to develop infectious uveitides, however, there is a gap in clear understanding of the mechanism for higher incidence of infections in the elderly and these individuals’ innate and adaptive immunity against infectious agents.

### Major Challenges in Ocular Infectious Inflammations-Detection of Unexpected and Undiscovered Infectious Agents

In infectious uveitides, there is overlap in the phenotypic expression of inflammations driven by various infectious agents. Thus, it is important to detect the infectious agent early to initiate proper interventions. An important challenge is that a biopsy of infected intraocular tissue used to detect the offending agent can be associated with vision threating complications. Thus, such tissue biopsies are rarely attempted in clinical practice of intraocular inflammations and infections. However, intraocular fluid analysis for cellular response, and detection of organisms by special strains, cultures and molecular techniques have been helpful in the diagnosis and treatment of infectious uveitides.

The microscopic examination of ocular fluids for detection of microbes is rapid and inexpensive but it requires expertise of laboratory technical personnel in staining the ocular fluids, as well as the expertise of professionals in interpretation of the morphology. Moreover, this approach has low sensitivity. In contrast, cultures of microbes provide high specificity and sensitivity but require a prolonged time to culture fastidious, acid-fast bacteria and fungi, leading to delayed diagnosis and interventions. Furthermore, culture methods are rarely used in detection of viral infections. Moreover, patients with prior use of antimicrobials can limit the growth of the organisms.

In over two decades above laboratory approaches are replaced by molecular techniques to detect infectious agents using intraocular fluids. The techniques include direct polymerase chain reaction (PCR), multiplex PCR, and targeted universal multiplex PCR. The later molecular techniques to detect microbes is based on employing universal primers for conserved 16S ribosomal RNA for bacteria. For detection of fungi, 18S rRNA and internal transcribed spacer (ITS) sequence are used. The universal multiplex PCR and other PCR approaches require presumptive diagnosis of etiologic infectious agents, these technique have low sensitivity with false positive and false negative results. Moreover, these amplification techniques are limited to a small portion of the microbial genome and are prone to contamination with environmental microbes. Furthermore, all the above molecular approaches are driven by clinical impressions to detect the infectious agent.

An important drawback of the above molecular approaches is the failure to detect unexpected and undiscovered organisms. This is a challenge in the field of infectious uveitides, however evolving techniques can address this challenge. By combining robust sequencing with metagenomics and bioinformatics (mNGS), a molecular approach could prove to be a sensitive and rapid method in the detection of both known and unknown infectious agents using a small sample of intraocular fluids. Moreover, since this approach is not clinical hypothesis driven, it is an unbiased technique in the diagnosis of bacteria, viruses, fungi, and other infectious agents. This offers the advantage of detecting currently known pathogens and previously unsuspected infectious agents. Moreover, mNGS can provide modeling of drug resistance and phenotypic behavior of pathogens (3). Potentially mNGS can be beneficial in targeted antimicrobial treatment, thus improving prognosis. Furthermore, big data based epidemiologic studies combined with mNGS can provide host susceptibility factors, prevention, and specific treatment of ocular infections prevalent in a country. Although mNGS is a robust technique in advancing the field of ocular infections and inflammations, its clinical adaptation may take time and requires proper validation by proven methods.

Usually, acute inflammation is self-limited in eliminating stimulus that initiates the inflammatory process. Persistence of the stimulus leads to chronic inflammation and also imbalance between the acute inflammatory process and pro-resolving mechanisms, which can result in persistent chronic inflammation. A challenge in infectious uveitis is the lack of understanding of the pro-resolving mechanisms. However, studies of inflammations unrelated to ocular tissues show that pro-resolving activity is an active process which includes specialized pro-resolving mediators (SPMS) that play a role in the resolution of acute inflammation and the prevention of the development of chronic inflammation (4). The SPMS’ role in resolving the inflammation is a complex one. Various ligands of G protein-coupled receptors, including lipoxins, resolvins, maresins and protectins have been identified. An understanding of SPMS and their mechanisms in elimination of ocular inflammation can lead to developing effective therapeutic interactions.

Although SPMS’ role in the resolution of inflammation is important, the elimination of noxious infectious agents is essential in preventing the development of chronic inflammation. Another process involved in the persistence of inflammation includes the NOD-like receptor family pyrin domain containing 3 (NLRP3) inflammasome, which is known to orchestrate innate immune response to infection. This multiprotein complex is activated through Caspase-1 with subsequent maturation of pro-inflammatory cytokines, pro-interleukin-1β and pro-IL-18. Such activation of cytokines offers protection in eliminating an infectious agent. However, there are knowledge gaps in the comprehensive understanding of NLRP3’s role in the persistence of ocular inflammation after eliminating the offending infectious agent. A clear understanding of NLRP3 assembly could determine the role of mitochondrial oxidative stress in tissue damaged and the controlling persistence of inflammation and autoimmunity.

### Major Challenges in Validating Disease Activity Biomarkers in Uveitis and Related Intraocular Inflammations

In humans, there are several reported clinical and ocular imaging findings in the management of uveitides. These include measurement of best-corrected visual acuity, anterior chamber cells and flare, vitreous haze, central macular edema, inflammatory retinal vascular wall sheathing, extent of choroidal and retinal lesions, and others (5). However, there has not been a robust validation of these findings in determining severity of uveitis during the active and resolving phases, in response to current therapeutic interventions and prognosis. Potentially surrogate biomarkers detected from blood and intraocular fluids can objectively address the status of active and quiescent uveitis, as well as the response to treatments and prognosis. Although detection of such biomarkers can be a challenge, current proteomic-based studies are revealing the presence of several hundreds of elevated proteins in the ocular fluids and blood. Similar ocular fluid and serum studies in various human uveitis entities and in animal models of uveitis show presence of various levels of cytokines and chemokines that are involved in the initiation or amplification of inflammation. Moreover, immunological studies reveal the importance of immunoreactive cells, particularly T cells in initiating and modulating an ongoing inflammatory process. Analysis of microRNA profiles in ocular fluid and blood samples may also uncover potential biomarkers of disease activity and response to therapy in uveitids patients.

It is recognized that modulation of the inflammatory process comes from the network and interaction of pro-inflammatory and anti-inflammatory cytokines. The former cytokines include members of IL-1, IL-6, TNFα, IL-17 and several members of IL-12, IL-23, and IL-39 families. The anti-inflammatory molecules include IL-10 family members, and IL-27 and IL-35. Although measurements of these cytokines in intraocular fluid and blood may yield promising results in active, resolving, and quiescent uveitides, studies are required to identify specific cytokines as biomarkers in various phases of uveitis.

Recent promising studies in animal models of uveitis and limited studies in humans with uveitis reveal that locally generated Th17/Th1 cells detected in intraocular fluid may serve as biomarkers for therapeutic responses. Presence of these cells was associated with resistance to corticosteroid treatment in suppressing the inflammation in uveitis. Clinical verification of the result from animal study will have significant impact in personalized/precision medical management of uveitis. The findings may be clinically significant by avoiding treatment of the uveitis with corticosteroids and corticosteroid related ocular and systemic complications (6).

The role of T regulatory cells (T-regs) in resolution of non-infectious uveitis and other autoimmune inflammations is well recognized. Adoptive transfer of T-regs can effectively bring about resolution and maintain remission of experimental autoimmune uveitis (7–9). The number and function of T-regs could be used to monitor or predict disease activity. Interestingly, Gilbert and colleagues have shown that the immune checkpoint molecule, T-cell immune receptors with Ig and ITIM domains (TIGIT), is critically involved in CD4^+^CD25^+^Fox P3^+^ T-regs-mediated resolution of uveitis (9). These TIGIT-expressing T cells may maintain clinical remission of the intraocular inflammation. In addition to T-regs, the immune checkpoint protein TIGIT is also expressed in activated and memory T cells, and NK cells (10). Enhancing the expression of immune checkpoint proteins such as TIGIT and cytotoxic T-lymphocyte antigen-4-immunoglobulin (CTLA4) using gene therapy or relevant fusion protein can be a novel therapeutic in recalcitrant uveitis of non-infectious etiology. It has been reported that the development of experimental autoimmune uveitis can be prevented by CTLA4 fusion protein (CTLA4-Ig) (11). The TIGIT-Ig fusion protein has been successfully used to treat murine model of systemic lupus erythematosus (12). Translation of the checkpoint protein results from animal studies into clinical practice remains to be a challenge.

Immune suppressive cytokines, such as IL-27 and IL-35 can be potential therapeutic targets. In uveitis animal models, recombinant IL-35 treatment resulted in suppression of the intraocular inflammation by inhibiting Th-17 responses and induction of regulatory B-cells (B regs). Interestingly IL-35 producing B regs (i35-B regs) generate abundant amounts of exosomes containing IL-35. Such exosomes can be potential immune suppressive therapeutic agents in treatment of uveitis (13). However, further studies are required to determine the role of the exosome-based therapy in human non-infectious uveitis.

The detection and validation of biomarkers as mentioned above including microRNAs in patients with various uveitis entities seem to be promising in guiding the development of novel personalized treatment options for uveitides. In patients with active and resolved uveitis, validation of microRNA changes in intraocular fluid and/or peripheral blood may also lead to the development of novel gene therapies that would avoid significant side effects of current treatments of uveitides.

## Inflammation-Related Ocular Degenerative Diseases

It is now well appreciated that inflammation plays a critical role in the pathogenesis of various ocular degenerative disorders such as DES, glaucomatous retinopathy, DR, and AMD. Research in the role of inflammation in ocular degenerative diseases is still in infancy and only limited results have been translated into clinical practice. The use of anti-inflammatory/immune suppressive drugs such as cyclosporine eyedrop has revolutionized DES therapy (14), although a significant proportion of patients do not benefit from the therapy. Currently, there is no FDA-approved immunotherapy for retinal degenerative diseases although there are compounds (e.g., complement inhibitors) in clinical trials. Further knowledge on detailed inflammatory pathways involved in the pathogenesis of ocular degenerative diseases is needed for the development of effective and safe immunotherapies. In the meantime, we also need clinical tools (e.g., biomarkers) to identify inflammation and such tools will be extremely valuable in implementing personalized medicine. A big challenge is to find disease-specific biomarkers and multiple “omics-based” approaches in combination with machine learning (ML) and artificial intelligence (AI) may help to overcome the challenge.

### Major Challenges in Uncovering the Pathogenesis of Ocular Inflammatory Diseases

Inflammation is a protective response of immune and non-immune cells against exogenous and endogenous pathogens and danger molecule, and has four phases, initiation, transition, resolution, and return to homeostasis. The collateral damage is often related to uncontrolled or dysregulated inflammation at the initiation and transition phases and the lack of proper resolution. Eliminating the initial triggers (e.g., with antibiotics or antiviral drugs for infectious ocular diseases) is most effective in controlling inflammation. In many conditions, it is a challenge to identify the factors that trigger inflammation. Therefore, therapeutic strategies rely on general immune suppression and the limited number of biologics that blocking inflammatory mediators. Improved knowledge on how inflammation is initiated and the mechanisms of immune dysregulation is essential to developing better therapies.

### Is Microbiota an Initiator and a Key Player of “Non-Infectious” Ocular Inflammatory Diseases?

Recent advance in microbiome studies has made us rethink the etiology of various non-infectious ocular inflammatory diseases. Microbiome encompasses trillions of microbes that exist in soils, oceans, plants, animals and human beings. The microbiome is an essential part of our biology that supports many physiological functions. For example, the gut microbiome is essential for our digestion and nutrition; it also helps to maintain the integrity of epithelial barrier and protects us from disease. The microbiome also presents in the ocular surface (15), and even inside the intraocular compartment (16). This ecosystem of commensal/pathogenic microbes is dynamic and influenced by the environment that we live, as well as our lifestyle, mental health, medication, and genetic background. A growing body of evidence suggests that gut and ocular microbial dysbiosis is critically involved in the development of intraocular inflammation, such as autoimmune uveoretinitis (15, 17, 18), AMD (19), and glaucoma (20). Furthermore, a recent study has reported disease-specific intraocular microbial signatures in eyes with senile cataract, AMD and glaucoma (16), suggesting possible infectious etiology of these age-related ocular diseases.

Microbial dysbiosis may contribute to the development and progression of inflammatory eye diseases by modulating the systemic immune system and/or altering the ocular microenvironment. Gut microbes and metabolites can be translocated into the bloodstream and peripheral tissues (21) and participate in the development of various autoimmune and neurodegenerative diseases. A low-grade systemic inflammation characterized by increased plasma levels of C-reactive protein, inflammatory cytokines (e.g., IL-1β, IL-6, TNFα), and neutrophil counts exists in various retinal degenerative conditions such as AMD and DR (22, 23), and this may result from gut microbial dysbiosis. The compositions of the ocular surface or intraocular microbiota may alter the ocular microenvironment and contribute to the development of ocular diseases. Establishing a causal link between specific commensal bacteria or their metabolites and ocular pathologies and the underlying mechanisms will be crucial to design better management strategies. Major challenges include the identification of disease-promoting microbiota in either the gut, ocular surface or intraocular compartments, and experimentally demonstrating their causal roles. It is very difficult to rigorously establish the causal role of individual microbes in human studies due to the extreme complexity of microbiota. Another challenge is the lack of proper models to study the causal role of individual microbes in human ocular inflammatory diseases. Although the gnotobiotic human microbiota-associated (HMA) mice are proven valuable in understanding the pathogenic roles of the gut microbiome in human disease, the bacteria compositions in the HMA mouse flora are not the same as the original donor fecal samples (24).

### Interaction Between Pathogens/Commensals and Ocular Barriers

The virulent pathogens do not normally have access to the intraocular compartment due to (a) the elimination of the pathogens by peripheral innate immunity, and (b) the blood-ocular barrier (BOB). The less virulent opportunistic pathogens can cross the blood-retinal barrier (BRB) and become latent (25). It is believed that pathogens may cross the BRB or blood-brain barrier (BBB) through the transcellular (e.g., receptor-mediated uptake or pinocytosis), paracellular or infectious cargo (e.g., infected monocytes) (25) although the detailed pathways involved in each route are poorly defined. It is unclear whether the commensals use the same routes to enter the intraocular compartments and how they influence the intraocular microenvironment. The commensals are detected in the aqueous humor and vitreous body in healthy eyes and they may come from the ocular surface and/or bloodstream through the cornea, iris, ciliary body, retinal blood vessels, and Bruch’s membrane/RPE barrier. The fact that there is a disease-specific intraocular microbiota profile (16) suggests a selection mechanism at BOB or within the intraocular microenvironment. We have found that RPE cells constitutively express high-levels of anti-microbial peptide lysozyme, which is critically involved in the bactericidal function of RPE cells (26). Our result suggests that, in addition to the physical barrier and immunosuppressive molecules, the BRB may also protect the neuronal retina from blood-borne pathogens by releasing antimicrobial peptides. It will be interesting to know what other antimicrobial peptides are produced by ocular barrier cells and how their production is regulated.

Once the ocular barrier is breached, retinal cells including neurons, glial cells and barrier epithelial cells (e.g., RPE) coordinate efforts to control infection. In that sense, retinal neurons [including photoreceptors (27)] and RPE cells have a strong immune suppressive and regulatory capability, which makes the retina an immune-privileged (IP) tissue. Although virulent pathogens may cause devastating intraocular inflammation, the opportunistic pathogens can persist as latent infection and later be reactivated when such immune protection is compromised. How the commensals affect the immune regulatory function of retinal cells remains unknown. The existence of disease-specific intraocular commensals suggests that they may play a critical role in modulating the retinal immune response in these degenerative diseases. To gain deeper insights into the pathogenesis of intraocular inflammatory diseases, a major challenge is to understand the complex communication between the microbiome, barrier cells (e.g., endothelial cells, ciliary body epithelial cells, and retinal pigment epithelial cells), neurons, and glial cells in homeostatic and disease conditions. This will require the establishment of suitable *in vitro* and *in vivo* models using various “omics-based” technologies. The investigations should cover genetic susceptibility, microbiome-ocular cell interaction, immune regulation, cell metabolism, etc. The application of ML and AI may help to model the complex network system of healthy, inflamed, or degenerative eye disorders.

### Aging and Ocular Inflammatory Diseases

Aging increases not only the risk of intraocular infection, but also various degenerative eye diseases, such as DES, AMD, and DR. There is a growing need to identify novel interventions to reduce the risk of age-related infectious and degenerative diseases by exerting a beneficial effect on the aging immune system. With advancing age, the immune system undergoes senescence (immunosenescence) leading to impaired ability to mount a robust immune response. On the other hand, age is accompanied by a low-level of systemic inflammation (inflammaging). The gut microbial composition changes drastically in old people characterized by the loss of protective commensals accompanied by the expansion of endotoxin-producing pathobionts (28). Such dysbiosis may contribute, at least partially, to inflammaging. On the other hand, a recent shotgun metagenomics study has shown that the gut microbiome of people with extreme longevity (aged 99 – 109y) is more suited for xenobiotic degradation and shows a rearrangement in metabolic pathways related to carbohydrate, amino acid, and lipid metabolism (29). A drastic difference exists in bacterial composition, metabolic functions, and the abundance of antibiotic resistance genes in ocular surface microbiome between young and old healthy donors (30). Whether these age-related changes contribute to DES remains unknown. It will be important going forward to investigate exactly how systemic and local immune responses are affected by dysbiosis in the elderly. The knowledge will offer opportunities to explore therapeutics approaches for mitigating microbial dysbiosis and restoring immune function in the elderly.

Although we do not know how age affects antimicrobial peptides production by ocular barrier cells, age impairs the blood-ocular barrier integrity and reduces the immune regulatory function of retinal neurons (31). This may increase the risk of dysregulated intraocular inflammation through multiple mechanisms. First, the weakened physical barrier allows easy access of circulating pathogens or commensals to the neuronal retina, hence, increases the risk of intraocular infection. Circulating immune cells (e.g., antigen presenting cells) can access retinal antigens, which are normally sequestered behind the blood-ocular barrier, through the impaired barrier, and this will increase the risk of autoinflammatory response. Second, the altered immune suppressive property of retinal neurons may lead to dysregulated or uncontrolled microglial or complement activation. Indeed, the aging retina is accompanied by a sustained low-level of inflammation (parainflammation) characterized by microglial activation, subretinal accumulation and low levels of complement activation (32).

The gut microbiome can affect the IP status of the CNS, including the retina and it is known that the gut microbiome in the early neonatal period is necessary for the establishment of the IP status of the CNS in adulthood. It would be interesting to know if the alterations in BRB and IP status in the elderly, particularly in patients with age-related retinal degenerative diseases, is related to unhealthy microbiome in infants or during aging.

We hope the *Frontiers in Ophthalmology – Inflammatory Eye Diseases* section will serve as a vehicle for discussion and exchanging opinions to synthesize and debate the grand challenges as well as publishing original research findings.

## Author Contributions

The writing of this manuscript was a collective effort between HX and NR. NR wrote the clinical part and HX wrote the basic science part of the challenges. All authors contributed to the article and approved the submitted version.

## Funding

The work of HX's lab is funded by Fight for Sight (UK) (1361/1362; 1425/1426; 5057/5018), Diabetes UK (11/0004230; 13/0004729; 16/0005537), the European Union's Horizon 2020 research and innovation program under the Marie Skłodowska-Curie (No 722717), Aier Eye Hospital Group Research Funds and Hunan Science & Technology Association (2018KX001; 2018RS3123).

## Conflict of Interest

HX has a sponsored research agreement with Boehringre Ingelheim and a consultant agreement with F. Hoffmann-La Roche Ltd.

The remaining author declares that the research was conducted in the absence of any commercial or financial relationships that could be construed as a potential conflict of interest.

## Publisher’s Note

All claims expressed in this article are solely those of the authors and do not necessarily represent those of their affiliated organizations, or those of the publisher, the editors and the reviewers. Any product that may be evaluated in this article, or claim that may be made by its manufacturer, is not guaranteed or endorsed by the publisher.
